# Signal Retrieval from Non-Sinusoidal Intensity Modulations in X-ray and Neutron Interferometry Using Piecewise-Defined Polynomial Function

**DOI:** 10.3390/jimaging7100209

**Published:** 2021-10-11

**Authors:** Simon Pinzek, Alex Gustschin, Tobias Neuwirth, Alexander Backs, Michael Schulz, Julia Herzen, Franz Pfeiffer

**Affiliations:** 1 Chair of Biomedical Physics, Department of Physics, School of Natural Sciences, Technical University of Munich, 85748 Garching, Germany; alex.gustschin@ph.tum.de (A.G.); julia.herzen@tum.de (J.H.); franz.pfeiffer@tum.de (F.P.); 2 Munich Institute of Biomedical Engineering, Technical University of Munich, 85748 Garching, Germany; 3 Heinz Maier-Leibnitz Zentrum (MLZ), Technical University of Munich, 85748 Garching, Germany; Tobias.Neuwirth@frm2.tum.de (T.N.); Alexander.Backs@frm2.tum.de (A.B.); Michael.Schulz@frm2.tum.de (M.S.); 4 Physik-Department E21, Technical University of Munich, 85748 Garching, Germany; 5 Department of Diagnostic and Interventional Radiology, School of Medicine, Klinikum Rechts der Isar, Technical University of Munich, 81675 München, Germany; 6 Institute for Advanced Study, Technical University of Munich, 85748 Garching, Germany

**Keywords:** grating interferometry, non-sinusoidal, intensity modulation, differential-phase contrast, dark-field contrast

## Abstract

Grating-based phase-contrast and dark-field imaging systems create intensity modulations that are usually modeled with sinusoidal functions to extract transmission, differential-phase shift, and scatter information. Under certain system-related conditions, the modulations become non-sinusoidal and cause artifacts in conventional processing. To account for that, we introduce a piecewise-defined periodic polynomial function that resembles the physical signal formation process, modeling convolutions of binary periodic functions. Additionally, we extend the model with an iterative expectation-maximization algorithm that can account for imprecise grating positions during phase-stepping. We show that this approach can process a higher variety of simulated and experimentally acquired data, avoiding most artifacts.

## 1. Introduction

**Figure 1:**
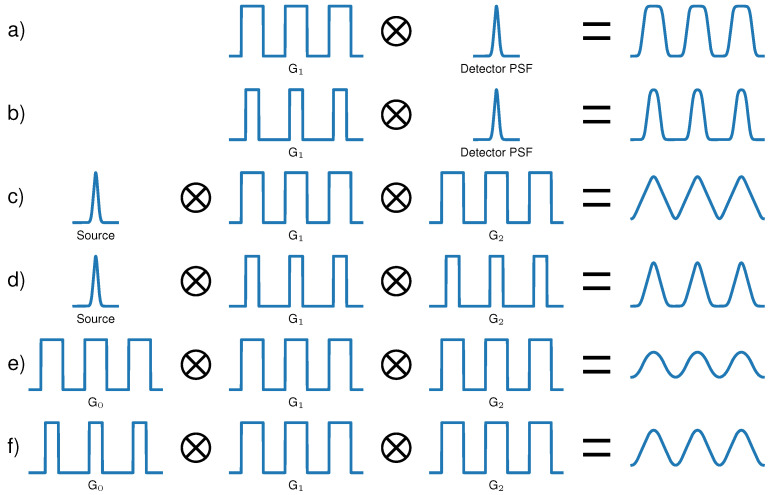
Origin of the resulting intensity modulation for different phase-sensitive imaging systems with periodic phase modulators. (**a**) Single grating system with a direct resolution of the intensity pattern can result in a trapezoidal modulation. (**b**) Single grating system with a smaller duty cycle (DC) resulting in an asymmetric modulation. (**c**) Two grating Talbot-interferometer operated with sufficiently coherent source resulting in a triangular modulation. (**d**) Two gratings with small DCs resulting in an asymmetric triangular modulation. (**e**) Three grating Talbot–Lau interferometer producing a quasi-sinusoidal modulation. (**f**) Talbot–Lau system with one grating having a small DC, resulting in deviations from sinusoidal modulations.

Conventional X-ray and neutron radiography methods exploit differences in the absorption of radiation between different materials. Apart from this well-established imaging method, other advanced contrast mechanisms have been developed, exploiting the wave properties of radiation [1,2,3,4,5,6,7,8,9,10,11]. Some of these methods use periodic X-ray and neutron optics (e.g., gratings, masks, compound refractive arrays) to modulate the beam’s intensity and measure its refractive and dispersive changes after transmitting an imaged object. The measured intensity modulation (IM) depends on the spatial and spectral properties of the used radiation source, the optics in the beam, the sample, and the used detecting system. Some methods use coherent synchrotron radiation [1,2,3,4,5,6] or micro-focus sources [10,11], but also low coherence sources such as conventional X-ray tubes [7,12] or neutron beamlines with relatively large pinhole apertures became applicable [8,9]. Some systems resolve the IM directly with high-resolution detectors [11,13], while others sample the periodic IM with an additional absorbing grating or mask in several exposures [7,8,10]. To model the blurring influence of a detector or an extended source and the relative motion between two gratings during acquisition, the observed IM can be calculated theoretically by the convolution of all gratings’ transmission profiles of the system [14], as illustrated in Figure 1. To extract an image of the sample-related interactions, a mathematical model of the IM based on the understanding of the physical properties of the system is desired. In most of the literature where 1D-sensitive systems (usually linear gratings or masks) are used, the IM is modeled by a simple sinusoidal function or a Gaussian, e.g., in aperture-based imaging [10,15]. However, there are many different reasons why the measured modulation could become a more complex function.

In the simple case of using a radiation source with high coherence, a single phase or absorption grating that creates a binary modulation (see Figure 1a), and a high resolution detector, the measured modulation can have a rather trapezoidal shape. This can be expected when, e.g., highly coherent, monochromatic synchrotron radiation is used. Further, the duty cycle (DC, ratio of transmitting area and period) of the generated profile can deviate from 0.5, resulting in an asymmetric modulation as shown in Figure 1b. This can happen when, e.g., apertures with narrow openings [10] or other kinds of non-binary gratings [16] are used that can generate a stronger sub-periodic focusing and result in advantageous aspects for system design. However, binary phase gratings with asymmetric DCs can also create high contrast modulations which are beneficial for system visibility [17].

If the IM cannot be resolved by a detector, a second grating can be used. Therefore, two rectangular intensity functions are convolved yielding a triangular IM. When a micro-focal source of sufficient coherence is used, this modulation is smoothed (see Figure 1c) but can still deviate quite strongly from a sinusoidal shape. In this case, the detector is usually recording a low frequency Moiré fringe pattern generated by both gratings, and the detected IM is decoupled from the Point Spread Function (PSF) of the detector. If the DCs of both gratings deviate remarkably from 0.5, the modulation becomes asymmetric (see Figure 1d). An example is the case of so-called edge-illumination setups where the absorbing apertures have relatively narrow openings such as 1/5 of the period [10]. When they are used with Gaussian-shaped sources, the final modulation can be well described with a Gaussian. In the case of ideal, coherent radiation, however, the modulation would be triangular.

Three grating interferometers such as illustrated in Figure 1e can be realized with absorption and phase gratings in different configurations. The source grating G${}_{0}$ is usually an absorption grating that exploits the Lau effect to achieve the needed coherence for the interferometer. Further beam shaping can be achieved with two absorption gratings [18], two phase gratings [19], or one phase and one absorption grating [7]. Since those systems usually operate with polychromatic spectra and consist of three optics, the resulting IM is well approximated with a sinusoidal function. However, if one or more gratings have DCs sufficiently smaller than 0.5, the modulation becomes non-sinusoidal, as shown in Figure 1f. Choosing smaller DCs for, e.g., source gratings, reduces the flux; however, it enables an increase in visibility. Chabior et al. [20] showed analytically that under simplified assumptions (e.g., perfectly absorbing gratings), the ideal DC of the source grating to reach the best signal-to-noise ratio in the phase image at a given flux is 1/3. Furthermore, when the source flux is not the limiting factor, but the absorbed dose as in medical applications, it is even more beneficial to tune the system towards higher visibility decreasing the DC of G${}_{0}$.

Further visibility increase can be achieved by optimizing the phase grating. The DC and phase shift of the latter can be adapted to improve its intensity focusing properties and performance with polychromatic spectra. Suleski [17] lists several configurations of binary phase gratings with DCs of 1/3 and 2/3 where compression ratios of 1:3 can be reached, and Chabior [14] evaluated possible system improvements in Talbot–Lau interferometers employing this principle. Similar effects of narrow intensity focusing as with conventional symmetric phase gratings can be also achieved with triangular, trapezoidal, and parabolic gratings. Using these different setup modifications to increase visibility, the IM can become non-sinusoidal and asymmetric with three-grating interferometers as well.

Revol et al. [21] showed that imprecise phase-stepping (’jitter’, deviation between assumed and actual grating positions during stepping) causes additional noise in all three image modalities. Conversely, similar noise is expectable when a sinusoidal modulation is fitted into non-sinusoidal stepping data since in both cases there is a discrepancy in the measured and assumed phase. Noise due to imprecise stepping can be avoided by, e.g., an iterative expectation maximization (EM) algorithm suggested by Wang and Han [22] and implemented for X-ray grating interferometry by Maschner et al. [23]. Another, faster approach based on principal component analysis was proposed by Vargas et al. [24] and adapted for X-ray grating interferometry by Pelzer et al. [25]. Several other combinations and modifications were discussed [26,27,28,29]; however, almost all literature treating 1D gratings assumes sinusoidal modulations. General algorithms which directly retrieve the phase shift by comparing small patches of a reference and a sample scan are used in, e.g., speckle-based X-ray imaging [30]. They can operate with periodic non-sinusoidal and even random IMs; however, they rely on directly resolving the shifted patterns and cannot handle interferometric modulations with large fringe sizes, which are typical for systems with multiple gratings.

Non-sinusoidal modulations have been approached by fitting higher orders of a Fourier series [31], which is a computationally inexpensive method for phase retrieval. However, it remains unclear how this approach can handle such a variety of modulations such as, e.g., given in Figure 1. Asymmetric modulations are potentially difficult to handle, as scattering not only reduces the amplitude but also changes the shape of the modulation. The more the IM shape deviates from a sinusoidal shape, the more additional parameters need to be fitted. Therefore, modeling the IM by a Fourier series with a higher number of orders introduces non-physical degrees of freedom, which could lead to over-fitting with a low number of phase steps.

To avoid those problems, we propose a more general model that consists of a polynomial piece-wise defined function, which results from convolutions of binary transmission profiles. A convolution of two binary profiles results in a triangular function consisting of linear contributions. A subsequent convolution of the triangular function with a third binary profile results in parabolic contributions. Hence, we propose a piecewise-defined function composed of zero to second order polynomials that can parameterize various combinations of convolved binary profiles. This function resembles the actual physical image formation process and is developed to cope with rectangular, trapezoidal, triangular, and sinusoidal IMs as well as asymmetric DCs such as the examples in Figure 1. As this piece-wise defined function requires more than three fitting parameters (as the sinusoidal function), we propose to determine several parameters related to the shape of the function from many sampling points measured in different pixels of the flat-field interferogram. Hence, this model contains the actual grating-related information of the system, which is not the case in previously discussed approaches. This way, the number of fitted parameters is reduced to four, while the individual shape of the modulation is taken into account at the same time. We compare and discuss the performance of the newly proposed model with simulated and experimental data.

## 2. Methods

### 2.1. Periodic Polynomial (PP) Intensity Modulation (IM) Function

To consider all possible shapes of the IM, a periodic polynomial (PP) function $I_{PP}$ with a period of 2π is introduced in the following, which is piecewise-defined of constant, linear, and 2nd order polynomials:

(1) $$I_{PP}\left(\phi \right)=\left\{\begin{array}{cc} I_{min}, & 0\;<|\phi -\Delta \phi |\leq \delta_{0}\\ a_{1}\times {\left(\phi -\Delta \phi -\delta_{0}\right)}^{2}+I_{min}, & \delta_{0}<\left|\phi -\Delta \phi \right|\leq \delta_{1}\\ m\times (\phi -\Delta \phi )+t, & \delta_{1}<\left|\phi -\Delta \phi \right|\leq \delta_{2}\\ -a_{3}\times {\left(\phi -\Delta \phi -\delta_{3}\right)}^{2}+I_{max}, & \delta_{2}<\left|\phi -\Delta \phi \right|\leq \delta_{3}\\ I_{max}, & \delta_{3}<\left|\phi -\Delta \phi \right|\leq \pi \end{array}\right..$$

**Figure 2:**
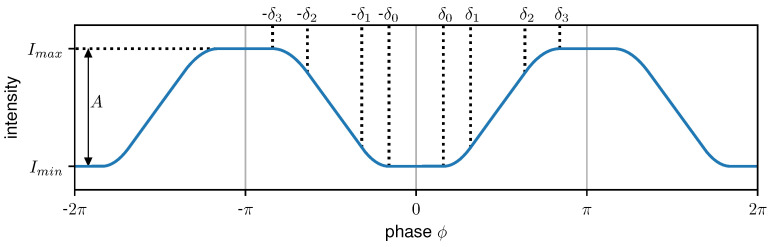
An example of the piecewise-defined periodic polynomial (PP) function $I_{PP}$ for Δϕ=0. Between 0 and $\delta_{0}$, respectively, $\delta_{3}$ and π$I_{PP}$ has a constant value of $I_{min}$ and $I_{max}$, respectively. For phase values between $\delta_{0}$ and $\delta_{1}$, respectively, $\delta_{2}$ and $\delta_{3}$$I_{PP}$ is parabolic while it is linear between $\delta_{1}$ and $\delta_{2}$.

Additionally, $\phi =2\pi xp^{-1}$ applies, where *x* is the spatial coordinate and ϕ is the corresponding phase as labeled on the horizontal axis in Figure 2. Δϕ is the phase position, $I_{min}$ and $I_{max}$ are the minimal and maximal intensity, respectively, and $\delta_{0}$ to $\delta_{3}$ indicate the transition points of the individual sections. From the additional boundary condition that $I_{PP}$ and $\frac{dI_{PP}}{dx}$ are continuous functions,

(2) $$m=2a_{1}\left(\delta_{1}-\delta_{0}\right),$$

(3) $$t=I_{min}-a_{1}\left(\delta_{1}^{2}-\delta_{0}^{2}\right),$$

(4) $$a_{3}=a_{1}\frac{\delta_{1}-\delta_{0}}{\delta_{3}-\delta_{2}},\;\mathrm{and}$$

(5) $$A=I_{max}-I_{min}=a_{1}\left(\delta_{1}-\delta_{0}\right)\left(\delta_{2}+\delta_{3}-\delta_{0}-\delta_{1}\right)$$

are derived, where *A* is the amplitude of $I_{PP}$. Combining Equations (1)–(5) $I_{PP}$ can be rewritten as a function depending on the seven free parameters: $\Delta \phi ,\;I_{min},\;A,\;\delta_{0},\;\delta_{1},\;\delta_{2},\;\delta_{3}$. Figure 2 shows an exemplary case for $I_{PP}$.

To reduce the number of parameters, $\delta_{0}$ to $\delta_{3}$ can be determined once in advance based on a flat-field acquisition and used again for further sample measurements with the same setup geometry. This is possible as the shape of the IM depends on the convolution of the individual optics and does not change between flat-field and sample acquisition. For this purpose, $I_{PP}\left(\phi ,\Delta \phi ,I_{min},A,\delta_{0},\delta_{1},\delta_{2},\delta_{3}\right)$ are fitted using a least-square (LS) optimization once to a dataset consisting of several pixels’ IMs from the entire field of view of the detector. In the following sections, 2500 pixels randomly distributed over the entire field of view of the detector were used to determine the shape for the simulated or measured dataset. Therefore, the stepping positions are shifted by the first-order phase determined by a sinusoidal processing for each pixel. Additionally, the intensities are scaled by the mean intensity of the respective pixel to compensate intensity gradients across the flat-field image. The determined values for $\delta_{0}$ to $\delta_{3}$ represent the shape of the flat-field IM. Then, the changes of intensity and phase by an object can be determined by fitting the three remaining parameters $\Delta \phi ,\;I_{min}$, and *A*. However, if the object induces small-angle scattering to the modulation, this not only leads to a decrease in visibility but also alters the shape of the IM. As a result, the shape of the IM is no longer adequately described by the previously determined values for $\delta_{0}$ to $\delta_{3}$. To account for this effect, the fitted flat-field intensity shape is convolved with several Gaussian kernels with index *i* and standard deviation $\sigma_{i}$. $I_{PP}$ is fitted again to each of the convolved intensity shapes to determine the corresponding transition points $\delta_{0,i}$ to $\delta_{3,i}$. Based on linear interpolation between these values, a scatter function SF can be created, which returns the corresponding transition points $\delta_{0}$ to $\delta_{3}$ for a certain scattering strength σ:

(6) $$SF\left(\sigma \right)=\left(\delta_{0},\delta_{1},\delta_{2},\delta_{3}\right).$$

Thus, an intensity shape can be assigned to a given scattering strength. Using Equation (6), $\delta_{0}$ to $\delta_{3}$ can be substituted by the scatter parameter σ, and the dependencies in $I_{PP}$ can be further reduced to

(7) $$I_{PP}\left(\phi ,\Delta \phi ,I_{min},A,\sigma \right),$$

where only $\Delta \phi ,\;I_{min},\;A,$ and σ remain to be determined by PP LS fitting. While the phase signal is directly given by the parameter Δϕ, the transmission *T* is determined by the mean of the intensity from a period

(8) $$T=\frac{1}{2\pi }\int_{-\pi }^{\pi }I_{PP}\left(\phi ,\Delta \phi ,I_{min},A,\sigma \right)\;d\phi ,$$

and the visibility is obtained from [12]

(9) $$V=\frac{I_{max}-I_{min}}{I_{max}+I_{min}}=\frac{A}{2I_{min}+A}.$$

### 2.2. Processing

In the following sections, two different PP processing algorithms are applied to the simulated and measured datasets and compared with two sinusoidal processing approaches. The first PP processing algorithm is the periodic polynomial least square (PPLS) fitting. This involves first determining the shape of the IM using 2500 randomly chosen pixels of the flat-field dataset to reduce the number of free parameters as described before. The number of 2500 pixels was chosen to take into account stepping data in all phase positions and to avoid over-fitting of the data. This is followed by fitting the stepping data of each pixel with $I_{PP}$. The PPLS is mainly compared to a conventional sinusoidal least square (SLS) fitting. As discussed in the introduction, even the minutest deviations in phase-stepping cause fringe artifacts and should be considered by optimizing the assumed phase (i.e., grating position) in every frame. Therefore, the second PP processing algorithm is a combination of the PPLS and EM, which in the following is called periodic polynomial expectation maximization (PPEM). The PPEM also determines the shape of the IM once at the beginning using 2500 randomly chosen pixels of the flat-field dataset. Then, $I_{PP}$ is alternately fitted to the stepping data of each pixel followed by an EM step where stepping jitter is corrected by minimizing the residual sum of squares (RSS). This is repeated until a previously defined convergence criterion is met. Here, the convergence criterion was set as the 99.9% percentile of the phase change between two iterations being less than 0.0001. The PPEM is mainly compared to a sinusoidal expectation maximization (SEM) processing.

### 2.3. Periodic Artifacts

As previously discussed, imprecise phase-stepping causes periodic artifacts in the retrieved images even with sinusoidal IMs. A similar effect is expectable for non-sinusoidal IMs even with an ideal phase-stepping, as the measured and theoretically expected shapes are different. Further complications are introduced as the non-sinusoidal modulation is sampled by a certain number of phase steps and different phases for every pixel. As a consequence, the retrieved values for transmission, visibility, and differential phase for a certain IM will slightly depend on the number of phase steps and at which phase the IM is sampled in the pixel.

Artifacts which originate from that are referred to as sampling artifacts. The period of these sampling artifacts is determined by the period of the intensity pattern in the acquired frames and the number of phase steps *N*. In case of equidistant phase steps, the resulting sampling artifact period is 1/N of the fringe pattern period as the measured sampling points repeat themselves after this distance (just shifted by a multiple of the phase 2π/N).

For evaluation and comparison of periodic artifacts, a Fourier analysis is performed in a region without a sample present. First, the mean value of the examined region of interest is subtracted as we are only interested in signal variations. Afterwards, a discreet Fourier transformation of all line profiles in the region of interest is performed and averaged. The frequencies are scaled in a way that the frequency of the fringe pattern $f_{0}$ corresponds to a value of 1. Note that the Fourier analysis is not a direct measure of how well the used model resembles the IM like the RSS. It is only used to compare the frequency and power of fringe artifacts between different processing algorithms for a certain set of parameters.

## 3. Simulation Results

### 3.1. Simulation of the Grating Interferometer Data

To evaluate the performance of the PP model compared to sinusoidal signal-retrieval, we simulated three exemplary systems with different numbers of gratings and grating DCs (see Table 1). The IMs for each system were numerically calculated by convolving binary grating profiles with the respective DCs. In case of the second and third system, an additional convolution with a Gaussian was performed to account for the source blur or the detector PSF. To evaluate the influence of phase-stepping, i.e., that the IM is sampled in different points in every pixel, the calculated IMs were translated into stepping data sets of a 300×200 pixel image array with five equidistant phase steps. The intensity values in the pixels were obtained by sampling the calculated IMs with different phases in a way that a Moiré fringe pattern with a period of 30 pixels occurs. Furthermore, a scattering and a phase object were placed in the simulated array to examine sample-related phase sampling artifacts for both evaluated processing schemes. On the left, a scattering object is placed. It consists of a gradient in scattering power from top to bottom and was calculated by convolving the IM with Gaussians of increasing σ from 0 to 0.4 times the period. On the right side of the simulated array, a sphere is placed as a pure, homogeneous phase-shifting object. Its phase shift was chosen in the lower range to visualize the interference of potential artifacts with the sample in a reasonable grey value windowing. Its differential phase shift was calculated by the derivative of its local thickness [6] resulting in Δϕ=±0.06π at the borders of the sphere.

As previously discussed, phase-stepping jitter introduced by hardware deficiencies is difficult to avoid and has a crucial influence on processing. To take this additional complication into account, we simulated stepping arrays where single frames were randomly shifted in phase. The phase deviations from ideal values were normally distributed with a standard deviation of 0.01π. With a 10 μm period grating, this would result in a ±50 nm displacement from the ideal positions, which is a realistic value for the precision of a medium quality linear stepping stage. Since the main objective of the work was to compare the different processing algorithms and investigate artifacts due to non-sinusoidal IMs, we did not add photon noise to the images.

### 3.2. Comparison of Sinusoidal and PP Approach for Interferometric IMs

**Figure 3:**
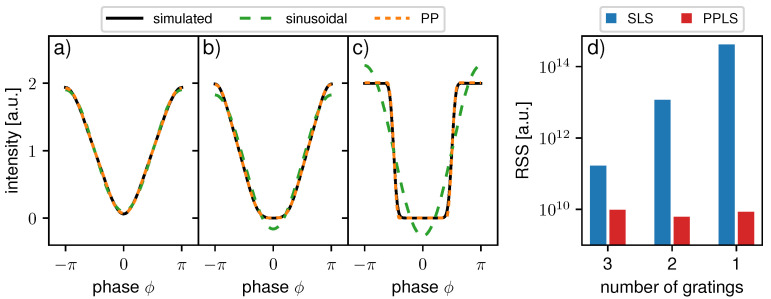
Simulated intensity modulations and fitted curves using a sinusoidal function and $I_{PP}$ for (**a**) a 3-grating interferometer, (**b**) 2-grating interferometer, and (**c**) 1-grating interferometer. (**d**) The residual sum of squares (RSS) of the flat-field processing for sinusoidal least square (SLS), and periodic polynomial least square (PPLS) of the entire dataset for the three interferometers in (**a**–**c**) simulated with 100 equidistant steps and no additional phase-stepping jitter.

For the first comparison of the sinusoidal and PP approach, the processing results of the three example systems without the influence of sampling-related artifacts are discussed. Therefore, we used 100 equidistant, precise phase steps for fitting, and the accuracy of the fits was evaluated by calculating the RSS on the entire dataset. Figure 3a–c shows a comparison between the simulated IM and fitted curves using both models. For the three-grating interferometer in Figure 3a, the sinusoidal, as well as the PP function, match the simulated IM well. For the two-grating interferometer, the linear part of the rather trapezoidal IM is fitted well; however, the minimal and maximal intensities are clearly underestimated by the sinusoidal model. As expected, the PP function resembles the shape of the modulation significantly better. Similar behavior is even more apparent in the third example in Figure 3c with a rectangular shape of the IM. The sinusoidal model overestimates the peaks and underestimates the minimum drastically, while the PP function reproduces the shape robustly. As observed in Figure 3d, the fitting accuracy represented by the RSS is much better for the PPLS than the SLS approach in all three exemplary systems. This was expected to some extent, since the PPLS optimizes one more free parameter than the SLS. However, the RSS of PPLS stays almost constant for sinusoidal, trapezoidal, and rectangular IMs, confirming its robustness for different shapes.

### 3.3. Periodic Fringe Artifacts Due to Sampling and Phase-Stepping Jitter

To visualize sampling-related artifacts introduced by the sinusoidal and PP approach, we simulated two datasets with five equidistant phase steps for the discussed two-grating system. The first one, with precise phase steps, was processed with SLS and PPLS. The second one was simulated with additional phase-stepping jitter. Hence, processing was conducted with SLS and PPLS but also with SEM and PPEM to address the imprecise phase-stepping positions.

#### 3.3.1. Simulated 2-Grating Interferometer with Precise Step Positions

**Figure 4:**
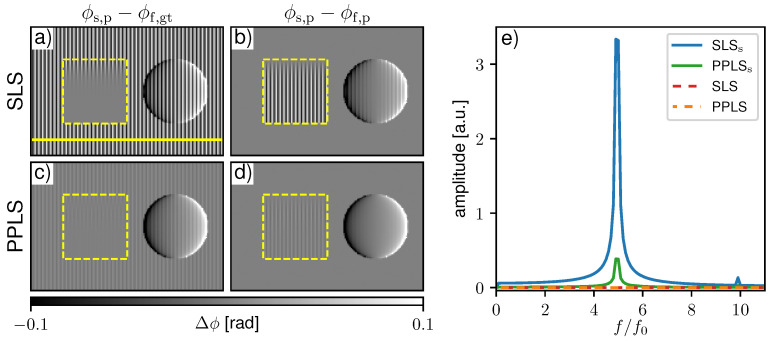
Comparison of processed differential-phase contrast (DPC) images with sinusoidal least square (SLS) and periodic polynomial least square (PPLS) fitting in case of precise phase steps. In (**a**,**c**) the sample image phase ($\phi_{s},p$) is processed with the ground truth flat-field phase ($\phi_{f},\mathrm{gt}$) to visualize sampling artifacts for the SLS and PPLS respectively. In (**b**,**d**) the DPC images are processed with fitted flat-field phase ($\phi_{f},p$), respectively. The yellow dashed squares mark the region with a scatter wedge with increasing scattering strength from top to bottom. Note that the images are windowed narrowly to visualize the fringe artifacts. (**e**) Corresponding Fourier spectra generated along the yellow line in (**a**).

To visualize sampling-related artifacts, the sample phase $\phi_{s,p}$ was processed with the ground truth flat-field phase $\phi_{f,\mathrm{gt}}$ in Figure 4a for SLS and c for PPLS. Respective differential-phase contrast (DPC) images processed with the fitted flat-field phase $\phi_{f,p}$ are shown in Figure 4b,d. SLS fitting shows pronounced fringe artifacts when processed with the ground truth phase, while PPLS processing reduces them significantly. This is also confirmed by the respective Fourier spectra in Figure 4e. When the image is processed with the fitted flat-field phase (Figure 4b), the background is flat since the sampling artifacts are compensated by identical phase biases. The same applies to PPLS fitting as observed in Figure 4c,d, as well as in the respective Fourier spectra. Within the scatter wedge in Figure 4a, the fringe artifacts disappear with increasing scattering strength since the IM transforms to a sinusoidal shape. When processed with fitted flat-fields (Figure 4b), the fringe artifacts appear where the dark-field (DF) signal is increased. This is a result of the biased flat-field and the artifact-free sample phase in the scattering object. Similar fringe artifacts are also present in the phase signal of the sphere in Figure 4b) since the phase in both the flat-field, as well as the sample image, is affected by sampling artifacts. Due to the improved fitting accuracy for non-sinusoidal IMs of the PP approach, in the respective PPLS image processed with the fitted phase (Figure 4d), the occuring artifacts in the scattering object are clearly reduced compared to the SLS.

#### 3.3.2. Simulated 2-Grating Interferometer with Phase-Stepping Jitter

**Figure 5:**
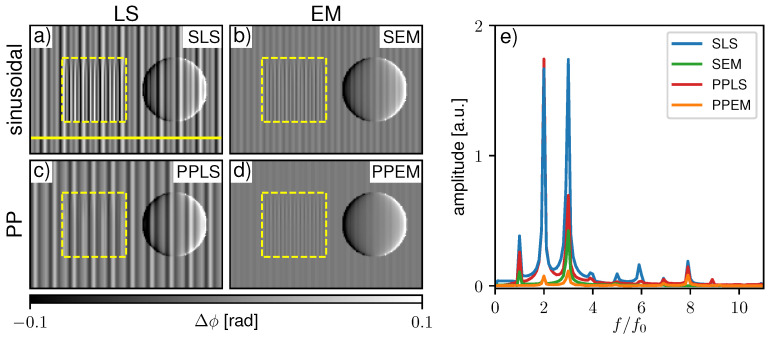
Processed differential-phase contrast images for (**a**) sinusoidal least square (SLS), (**b**) sinusoidal expectation maximization (SEM), (**c**) periodic polynomial least square (PPLS), and (**d**) periodic polynomial expectation maximization (PPEM) of the simulated two-grating interferometer with phase-stepping jitter. The yellow dashed squares mark the region with a scatter wedge with increasing scattering strength from top to bottom. (**e**) Corresponding Fourier spectra generated along the yellow line in (**a**).

The stepping process is usually not precisely reproducible, and one cannot rely on similar deviations in sample and flat-field acquisition. Thus, one cannot expect that sampling artifacts would cause similar phase biases, which would be compensated as in the previous example with precise phase steps. Figure 5a–d show processed DPC images of the same system as before with additional phase-stepping jitter as described in Section 2.2. All images were processed with fitted flat-fields, which were affected by jitter as well. Conventional sinusoidal LS fitting (Figure 5a) shows a much more complex fringe artifact pattern as different types of artifacts are superimposed. When SEM processing is applied (Figure 5b), a high fraction of the artificial frequencies is removed compared to SLS as the Fourier spectra show in Figure 5e. Even though PPLS processing considers the non-sinusoidal shape of the IM, it cannot reduce the fringe artifacts (Figure 5c) when phase-stepping jitter is not corrected for. Most accurate results are obtained by PPEM processing (Figure 5d), where PP fitting is performed with an EM-based correction for imprecise stepping positions. Hence, fringe artifacts due to non-sinusoidal IMs and related sampling artifacts should be distinguished from phase-stepping jitter and corrected separately if necessary.

**Figure 6:**
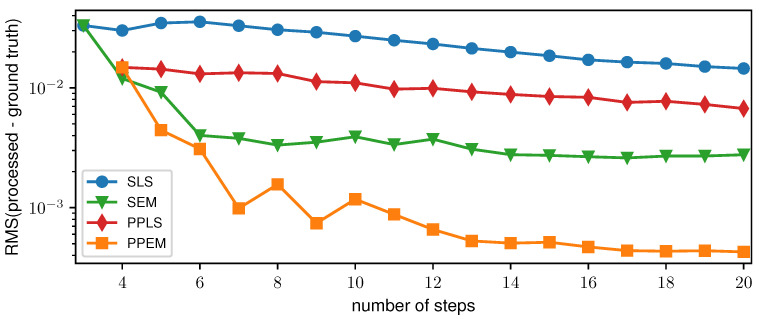
Root-mean-square (RMS) of the difference between the processed and ground truth differential-phase contrast images of the 2-grating interferometer over the number of phase steps for all four processing algorithms.

To evaluate the performance of the PP approach depending on the number of phase steps, the root-mean-square (RMS) of the difference between the processed and ground truth DPC images of the two-grating interferometer was determined. Ten datasets for each number of phase steps were simulated and processed in the range from three (SLS and SEM) and four (PPLS and PPEM), respectively, and up to 20 phase steps. The averaged RMS for each number of phase steps and each processing algorithm is displayed in Figure 6. For SLS and PPLS, a slow decrease in the RMS can be seen, whereas the PPLS is always below the SLS. SEM and PPEM show a steep decrease for small numbers of phase steps which flattens for larger numbers of phase steps. For five or more phase steps, the RMS of the PPEM is below the SEM and therefore performs superiorly in terms of sampling artifact reduction.

## 4. Experimental Results

Two different experimental examples of X-ray and neutron grating-based imaging are presented to test the newly proposed processing model. To demonstrate that the PP approach also leads to a reduction of sampling-related artifacts on experimental data, we processed an X-ray and a neutron dataset using the SLS, SEM, PPLS, and PPEM.

### 4.1. Data Acquisition

**Figure 7:**
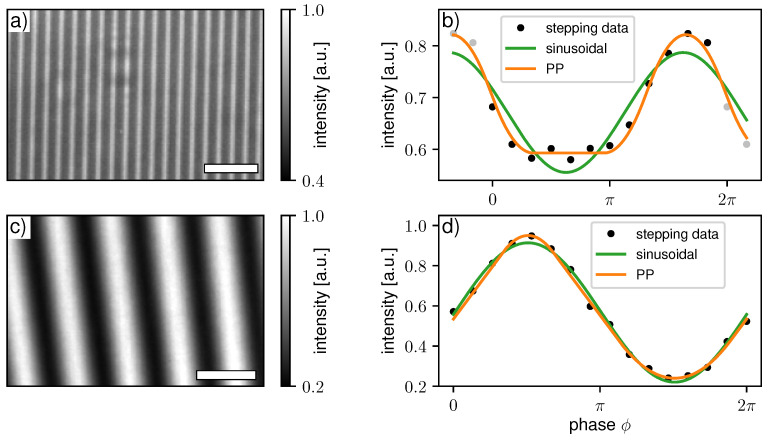
Detector image of a flat-filed intensity modulation for the (**a**) 1-grating X-ray interferometer and (**c**) 3-grating neutron interferometer. Measured stepping data and fitted curves using a sinusoidal function and $I_{PP}$ of a single detector pixel for the (**b**) one-grating X-ray interferometer and (**d**) three-grating neutron interferometer. For a better visualization in (**b**), the stepping data were periodically continued in both directions (gray markers). The white scale bars in (**a**,**c**) represent 20 μm and 10 mm, respectively.

**Figure 8:**
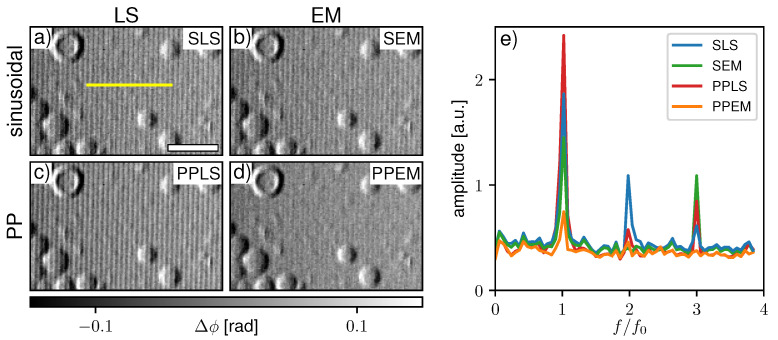
Differential-phase contrast images processed by (**a**) sinusoidal least square (SLS), (**b**) sinusoidal expectation maximization (SEM), (**c**) periodic polynomial least square (PPLS), and (**d**) periodic polynomial expectation maximization (PPEM) from a single grating X-ray interferometer. (**e**) Fourier spectra for the respective processing schemes averaged from 50 pixel rows next to the yellow line in (**a**). The white scale bar in (**a**) represents 50 μm.

First, a single phase-grating setup with an asymmetric DC is discussed. The scans were acquired at the beamline P05 at Petra III/DESY (Hamburg, Germany) [32] using highly coherent X-ray synchrotron radiation generated by an undulator source and monochromatized by a double crystal monochromator at 15 keV. The silicon grating (period: 5 μm, DC ≈ 0.33, height: ≈25 μm) had a binary profile fabricated by anisotropic potassium hydroxide etching. It acted as a 4/3π shift array, producing an asymmetric modulation at around 1/6 of the Talbot distance. The diffraction pattern was directly recorded by a high-resolution CMOS camera that was focused on a 50 μm thick lutetium–aluminium garnet (LuAG) scintillator with 10× optical magnification, resulting in a pixel size of 0.64 μm. In total, 12 phase steps ranging over one period were acquired with an exposure time of 200 ms per frame for the sample and the flat-field scan. A detector image of the flat-field scan and the stepping data of a single pixel (Figure 7a,b) reveal the non-sinusoidal nature of the measured IM clearly. The sample shown in Figure 8 consists of full and hollow silica (SiO${}_{2}$) spheres with several tens of μm in diameter.

**Figure 9:**
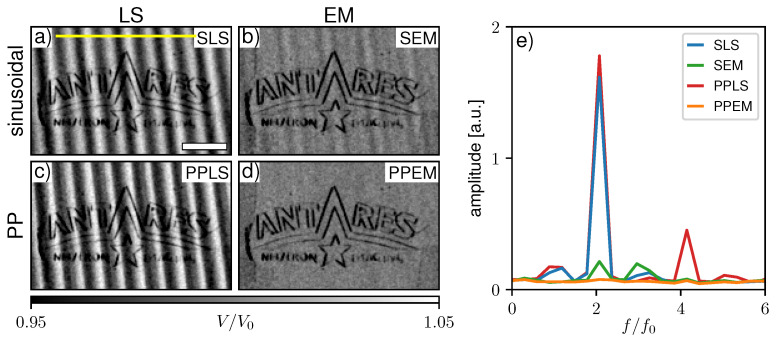
Dark-field images processed by (**a**) sinusoidal least square (SLS), (**b**) sinusoidal expectation maximization (SEM), (**c**) periodic polynomial least square (PPLS), and (**d**) periodic polynomial expectation maximization (PPEM) for the dataset measured at the Antares beamline. (**e**) Fourier spectra for the four algorithms averaged about 60 pixel lines along the yellow line in (**a**). The white scale bar in (**a**) represents 10 mm.

The second example is a three-grating Talbot–Lau interferometer implemented at the ANTARES beamline at FRM II (Garching, Germany) [33]. The setup was operated with a neutron velocity selector providing quasi monochromatic neutron radiation of 4 Å (Δλ/λ=10%). Unlike typical Talbot–Lau systems operating with laboratory X-ray sources with polychromatic spectra, it is to expect that the modulation produced by the G${}_{1}$ is rather more rectangular than sinusoidal. Furthermore, a source grating G${}_{0}$ with a DC of 0.3 was used to increase the visibility and the DC of the G${}_{2}$ was 0.45 due to fabrication-related deficiencies. The interferometer geometry is asymmetric to keep the distance of the sample to the detector as low as possible and avoid geometric source blur. The period of the G${}_{1}$ grating is 24.4 μm, and the linear stage used for its stepping (MFA-PPD, Newport Corporation, Irvine, US) has a typical bi-directional repeatability of 0.2 μm according to the manufacturer. In this example, a phase stepping with 16 steps each with 20 s exposure time was performed. A detector image of the flat-field scan and the stepping data of a single pixel (Figure 7c,d) reveal a slight deviation of the measured IM from a sinusoidal shape. We found that an EM-based determination and correction of the stepping positions was necessary in this case as well to avoid fringe artifacts. The imaged sample (DF modality shown in Figure 9) was a logo of the ANTARES beamline engraved into an aluminum sheet. Further details on the geometry, grating properties, used hardware and overall performance of the instrument can be found in [34,35].

### 4.2. Data Processing

#### 4.2.1. Phase Retrieval from a Single Grating X-ray Interferometer

Figure 8a–d show a comparison of the four previously discussed processing schemes with experimentally acquired data by the single phase grating X-ray interferometer. The sample is a substrate with hollow and filled silica micro-spheres. Respective Fourier spectra along the yellow line in Figure 8a are given in Figure 8e. Since the IM created by the system is asymmetric and non-sinusoidal, strong fringe artifacts appear in SLS processing (Figure 8a). SEM (Figure 8b) and PPLS (Figure 8c) fitting hardly reduce the artifacts, which implies that both phase-stepping jitter and non-sinusoidal IMs are present. Only considering both with the PPEM processing (Figure 8d) brings a clear improvement. Some residual fringes still remain as the Fourier spectrum (Figure 8e) shows; however, they are less pronounced compared to the background noise level.

#### 4.2.2. Dark-Field Signal from a Neutron Talbot–Lau Interferometer

As already observed in Section 3.2, the amplitude of non-sinusoidal IMs is not fitted accurately by the sinusoidal models even with a high number of sampling points. Hence, with a low number of phase steps it is assumed that the fitted amplitude will be over- and underestimated in different pixels, leading to similar sampling-related fringe artifacts in the DF modality as in the DPC images. Figure 9a–d compare the previously discussed processing schemes in the DF modality acquired with the neutron Talbot–Lau interferometer. The scan is strongly affected by phase-stepping jitter as SEM processing (Figure 9b) brings a drastic improvement compared to SLS (a) and PPLS (c). However, the SEM image and the respective Fourier spectrum (e) show some faint fringe artifacts. The PPEM (d) again shows the best result, eliminating the fringes by considering both phase-stepping jitter and non-sinusoidal IM.

## 5. Conclusions

We introduced an alternative polynomial piece-wise defined function which allows to model IMs, reproducing their physical origin from several convolutions with binary intensity profiles. The PP function can handle periodic symmetric and asymmetric IMs from interferometric and non-interferometric origin recorded in a phase-stepping process. We simulated imaging systems with 1–3 gratings with different DC and compared the performance of the PP approach with conventional sinusoidal processing using LS fitting. We showed that sampling-related fringe artifacts in the DPC and DF images can occur when non-sinusoidal IMs are processed with conventional sinusoidal models. Those are similar to phase-stepping jitter artifacts; however, they have a different origin. To distinguish both, we employed an EM algorithm that uses the entire interferogram to correct for phase-stepping positions and combined it with the sinusoidal (SEM) and PP (PPEM) approach. We found in simulation that even small deviations (jitter) from the assumed phase steps create more severe artifacts than the discrepancies between the non-sinusoidal IM and the sinusoidal assumption. For acquisitions with five and more phase steps, the PPEM performs superior to the SEM since it successfully corrects for jitter and almost completely eliminates residual fringe artifacts originating from non-sinusoidal IMs. Consistent behavior is found in DPC and DF images for the experimental data acquired with a single grating and a Talbot–Lau interferometer.

We conclude that grating-based imaging methods operated with multiple gratings and highly coherent sources (e.g., small source spots, monochromatic radiation) or high-resolution detectors are prone to non-sinusoidal IMs, which should be considered in image processing. Especially when source gratings with small DC or triangular phase gratings that create an asymmetric modulation are used, a thorough analysis and modeling of the IM should also be performed with three-grating interferometers. EM-based algorithms or similar alternatives should be used in any case to correct for phase-stepping deficiencies. Residual fringe artifacts can have their origin in non-sinusoidal IMs and can be addressed with the proposed PPLS or PPEM approaches. For that, high quality flat-fields with many phase steps should be acquired to determine the system-related fitting parameters. Spatial variation of the grating parameters (e.g., DC) might require determining the shape parameters pixel by pixel. The sample measurement can be conducted with fewer steps in dose-relevant applications. Another crucial aspect not evaluated in detail is the flux stability of the source. In case of fluctuations, a respective monitoring or advanced flux estimation must be included in the model.

The proposed PPEM approach allows to process a high variety of data from interferometric and non-interferometric systems. Further, it is adaptable to different grating-related circumstances that have not been addressed before. However, it comes at the cost of high computational effort since piece-wise defined functions with many parameters must be optimized iteratively. Apart from X-ray and neutron grating interferometry, the PPEM can be used for many other applications where phase retrieval from non-sinusoidal Moire-patterns is needed.

## Figures and Tables

**Table 1:** System parameters for the three simulated systems. The source size or detector Point Spread Function (PSF) is a Gaussian and its standard deviations σ are given in multiples of the period.

System	Source/Detector PSF σ	G${}_{0}$ DC	G${}_{1}$ DC	G${}_{2}$ DC
3-gratings	-	0.2	0.5	0.4
2-gratings	0.05	-	0.333	0.4
1-grating	0.02	-	0.5	-

## Data Availability

Data underlying the results presented in this paper are not publicly available at this time but may be obtained from the authors upon reasonable request.

## Abbreviations

The following abbreviations are used in this manuscript:

DC	duty cycle
DF	dark-field
DPC	differential-phase contrast
EM	expectation maximization
IM	intensity modulation
LS	least-square
PP	periodic polynomial
PPEM	periodic polynomial expectation maximization
PPLS	periodic polynomial least square
PSF	point spread function
RMS	root-mean-square
RSS	residual sum of squares
SEM	sinusoidal expectation maximization
SLS	sinusoidal least square
